# Costing Hospital Surgery Services: The Method Matters

**DOI:** 10.1371/journal.pone.0097290

**Published:** 2014-05-09

**Authors:** Gregoire Mercier, Gerald Naro

**Affiliations:** 1 CHU de Montpellier, Montpellier, France; 2 Montpellier Research in Management, Universite Montpellier 1, Montpellier, France; National Institute for Public Health and the Environment, Netherlands

## Abstract

**Background:**

Accurate hospital costs are required for policy-makers, hospital managers and clinicians to improve efficiency and transparency. However, different methods are used to allocate direct costs, and their agreement is poorly understood. The aim of this study was to assess the agreement between bottom-up and top-down unit costs of a large sample of surgical operations in a French tertiary centre.

**Methods:**

Two thousand one hundred and thirty consecutive procedures performed between January and October 2010 were analysed. Top-down costs were based on pre-determined weights, while bottom-up costs were calculated through an activity-based costing (ABC) model. The agreement was assessed using correlation coefficients and the Bland and Altman method. Variables associated with the difference between methods were identified with bivariate and multivariate linear regressions.

**Results:**

The correlation coefficient amounted to 0.73 (95%CI: 0.72; 0.76). The overall agreement between methods was poor. In a multivariate analysis, the cost difference was independently associated with age (Beta = −2.4; p = 0.02), ASA score (Beta = 76.3; p<0.001), RCI (Beta = 5.5; p<0.001), staffing level (Beta = 437.0; p<0.001) and intervention duration (Beta = −10.5; p<0.001).

**Conclusions:**

The ability of the current method to provide relevant information to managers, clinicians and payers is questionable. As in other European countries, a shift towards time-driven activity-based costing should be advocated.

## Introduction

Healthcare reforms worldwide have led to an increased reliance on hospital accounting practices. In a resource-constrained environment, accurately estimating the cost of hospital services is of the utmost importance in the pursuit of efficiency and transparency. Hospitals are financed through DRG (Diagnosis Related Group)-based prospective payment systems in most high-income countries [1]. In this context, hospitals have to locate and eliminate inefficiencies, i.e., services for which the production cost is significantly higher than the price [2]. Hence, hospitals need reliable patient-level cost estimates to accurately measure resource utilisation [3], [4], [5]. Accurate and relevant cost information on hospital services at the patient level is therefore fundamental for policy makers, payers and hospitals.

However, costing is particularly difficult in the hospital setting due to case heterogeneity, labour intensity and the complexity of the production processes. Evidence shows considerable cost variation for a given service which can result from provider and patient characteristics, the efficiency level, the underlying clinical activity and, most importantly, the costing method [6].

Allocating hospital costs to patients (or groups of patients) typically involves three steps [7]: the allocation of hospital overhead costs to departments, the allocation of department overhead costs to patients, and the allocation of department direct costs to patients. The focus of this work is on the third step. Indeed, various costing methods are used to allocate department direct costs to patients, and there is a lack of standardisation [8], [9]. A two-step classification process of hospital costing methodologies based on the level of accuracy has been proposed [10]. First, cost components are identified either at the aggregate level (gross costing) or at the patient level (microcosting). Then, cost components are valued either by allotting costs from comprehensive sources (top-down approach), or by identifying resource consumption at the patient level (bottom-up approach). According to this classification, top-down microcosting results in average unit costs per patient, whereas bottom-up microcosting leads to patient-specific unit costs. Countries apply either method, mainly depending on the availability of patient-level cost data. For instance, Finland, Germany, the Netherlands and Sweden apply a bottom-up approach, while England, Estonia and France rely on a top-down method [7].

All other things being equal, the choice of costing method might significantly affect the cost estimates [10], [11], [12]. Few studies have investigated the agreement between bottom-up and top-down microcosting on large samples, which seems to be moderate at best [13]. In the hospital setting, bottom-up microcosting is considered to be more accurate and relevant than top-down methods [5], [7], [10]. It has been recommended to apply a bottom-up microcosting method for hospital services having a large proportion of labour and overhead [14]. However, identifying costs at an individual patient level is time consuming and expensive [14]. It is therefore advocated to restrict the use of bottom-up microcosting for the cost components having a significant impact on the total cost, namely labour costs and labour-intensive services [10]. In the context of international clinical trials, different methods might lead to different estimates of average treatment costs [15], [16], [17], [18].

In France, the current costing method for surgical services is top-down microcosting, which consists of allocating aggregate costs to individual operations according to the surgical procedure performed on a work unit basis called relative cost index (RCI) [19], [20]. Consequently, a given procedure performed in a given setting will always be allocated the same cost. Intuitively, the main limitation of this approach lies in the fact that it ignores the variation in resource consumption occurring for the individual patient, as reflected for instance by the variation in procedure durations or by the variation in the number of medical and non-medical personnel involved.

Activity-based costing (ABC) is a well-known bottom-up microcosting method that has been used in the service sector since the 1980s [21] and, more recently, to cost surgery services [13], [22], [23], [24], [25], [26]. ABC estimates the cost of individual hospital services by assessing the actual amount of specific resources that contribute to produce each service. In contrast to top-down microcosting, activity-based costing accounts for the individual within-procedure variation in resource use. Similarly to other bottom-up microcosting methods, its main limitations lie in the complexity and cost of implementation, which might explain a low adoption rate in the hospital sector [27].

This study focuses on the costing of abdominal surgery procedures for two reasons. First, it seems reasonable to assume that surgery procedures are among the most labour-intensive healthcare services. Second, surgical care has been shown to exhibit the lowest agreement between top-down and bottom-up cost estimates [13].

The aims of the study were (1) to assess the agreement between the two costing methods and (2) to identify variables cost differences.

## Materials and Methods

### Setting and Data

The study took place in the abdominal surgery department of the Montpellier University Hospital, a French 2 150-bed tertiary centre. All consecutive inpatient surgical procedures performed during the year 2010 in this department were included. Total hospital expenses incurred from the entry into the operating room until the exit from the post-anaesthesia care unit were considered, including those related to the preparation, cleaning and management of the operating theatres. Three categories of expenses were considered and provided by the hospital accounting department: (i) medical, nurse and administrative staff, (ii) drugs and medical equipment (including depreciation and maintenance) and (iii) overhead costs (supplies, taxes, insurance, utilities and loan interest). Prostheses and other single-use materials were not considered, as our purpose was to focus on expenses that are potentially subjected to allocation errors. Data on resource use in the operating room were provided by a computerised operating room register. Individual unit costs were estimated twice at the patient level, using the current top-down method and the bottom-up method. Clinical information was extracted from electronic medical records. The accounting database, the operating room register and medical records were linked using an anonymous patient ID. Using such information for research purposes is allowed by French law.

### Top-down Microcosting

Individual unit costs for the same sample of surgical procedures were estimated with the current top-down microcosting method. The calculation followed the national guidelines issued by the French Ministry of Health [28]. The top-down method involves two steps. First, staff costs are allocated to each procedure proportionally to the pre-defined cost weights. Indeed, the French classification of medical procedures is similar to the ICD-10 Procedure Coding System and assigns a relative weight to each surgical procedure called a Relative Cost Index (RCI). RCIs were defined by medical expert panels in the early 1990s [29] and were revised in 2003 [30]. They are updated yearly to incorporate new procedures. RCIs ought to reflect the average resource consumption intensity attached to each surgical procedure and should aim to cover all direct and indirect costs excepting overheads. This calculation is made irrespective of the day (weekday vs. weekends/nights) and of the actual duration and staffing level of a given operation. The second step consists of allocating the remaining costs (drugs and medical equipment and overheads) proportionally to the staff costs, and therefore, proportionally to the RCI as well. Altogether, the top-down microcosting method relies solely and entirely on pre-determined cost weights (RCIs).

### ABC Bottom-up Microcosting

The implementation of the ABC model followed three standard steps: mapping activities, calculating the cost of each activity and calculating the unit cost of each procedure. The activity mapping consisted of first systematically analysing the standard operation procedures and then performing semi-structured interviews with administrative, nurse and medical staff of the surgery and intensive care wards. Eleven relevant, mutually exclusive and collectively exhaustive activities were defined. Seven primary activities matched the chronological steps of performing a surgical procedure, from the admission of the patient into the operating room until their exit from the post-anaesthesia care unit. These activities included the individual operating room setup, the patient’s positioning on the operating table, the induction of anaesthesia, the surgical procedure itself, the wound dressing, the cleaning and the recovery time in the post-anaesthesia care unit. Additionally, four secondary activities were defined: daily operating room setup and cleaning, planning management, surgical management and anaesthetic management. Management activities included all other tasks performed by the surgery or anaesthetic staff but unrelated to a given patient. Activity costs were calculated using cost drivers available in the hospital information system: staffing levels and duration of each step. The computerised operating room register provided information on the number of professionals involved in each step of a given individual procedure and on its duration. The average time spent by administrative, nurse and medical staff on secondary activities was assessed through semi-structured interviews. To account for the actual staffing practices, the calculation distinguished between anaesthesiology and surgery staff as well as between weekdays, nights or weekends. By weighting the cumulated duration of each activity with its staffing level, we calculated the total staff cost of each activity. All non-staff costs, including overheads and equipment amortisation, were added proportionally to staff costs. Unit costs per individual patient were finally computed by linking each procedure to primary and secondary activities through actual durations, actual staffing levels and the total number of procedures. This calculation was performed consecutively for anaesthetic and surgical costs and separately for weekdays and weekend/night procedures.

### Statistical Analysis

Unit costs were presented using means, standard deviations and quartiles as appropriate. The overall agreement between the two methods was assessed using two complementary approaches: the Spearman non-parametric coefficient of correlation and the Bland and Altman method [31]. Regarding the correlation coefficient, values above 0.5 are deemed satisfactory for group comparisons (i.e., comparisons of several strategies based on pooled data from multiple patients) and values above 0.9 are adequate for individual assessments (i.e., the cost estimation for a single patient) [13]. The Bland and Altman method is widely used in clinical epidemiology when the same concept is measured by two different methods. The acceptability threshold was set at 2 000 Euros, i.e., the average unit cost per inpatient stay at Montpellier University hospital in 2010. The cost difference between the two methods was calculated as top-down cost minus bottom-up cost. Hence, a positive cost difference indicates that the top-down cost is greater than the bottom-up cost. To understand discrepancies, a bivariate analysis of the cost difference was performed using linear regression models with the following variables: gender, age, anaesthetic risk, staffing level (number of surgeons performing the procedure), status of the responsible surgeon (senior vs. junior surgeon), duration of the intervention phase, relative cost index (RCI), occurrence of an anaesthetic complication during the procedure, emergency context and procedure performed during on call periods (weekends or nights). The individual anaesthetic risk was assessed through the American Society of Anaesthesiologists score (ASA), which measures the physical status of patients before surgery [32]. The ASA score ranges from 1 (full health) to 6 (brain-dead patient). Age, ASA score, RCI, staffing level and intervention duration were analysed as continuous variables. Then, a multivariate analysis of the cost difference was implemented using a multiple linear regression model. Only variables significantly associated with the cost difference in bivariate analysis were included in the multivariate model. All statistical tests were performed using a 5% type-one risk. Statistical analyses were performed with the statistical software SAS version 9.2 (SAS Institute, Cary, N.C.).

## Results

Between January and October 2010, 2 943 consecutive procedures were performed. Of these, 813 (28%) were non-surgical procedures (central venous catheter placements, resuscitation procedures and endoscopies) and were therefore included in the costing process but excluded from the statistical analysis. These very short and low-cost procedures would have unnecessarily increased the data heterogeneity. The statistical analysis was conducted on a sub-sample of 2 130 surgical procedures, of which 422 (19.8%) had been performed in an emergency context and 238 (11.2%) had been performed during weekends or nights (Table 1). Patients were 54 years old on average, and 975 (46%) were female. The total cost of the 2 130 surgical procedures was provided by the accounting department as 4 962 900 Euros, of which 56% was for non-medical staff, 23% for medical staff, 16% for drugs and medical equipment and 5% for overheads. The relative cost index (RCI) of the 2 130 procedures ranged from 14 (minor superficial surgery under local anaesthesia) to 1 293 (total hepatectomy). According to the top-down method, the average cost per individual procedure was 2 331 Euros (SD = 1 720) and ranged from 79 to 16 721 Euros (Table 1). According to the ABC bottom-up method, primary activities amounted to 87% of the total expenses (Table 2). The average cost per individual procedure was 2 186 Euros (SD = 1 391) and ranged from 648 to 14 516 Euros (Table 1). Table 2 shows detailed duration, cost driver, unit costs and total cost per activity.

**Table 1:** Sample characteristics.

	N = 2 130
Age (years)	54 (SD: 18)
Female patient	975 (45.8%)
Junior surgeon	56 (2.6%)
On-call period	238 (11.2%)
Emergency	422 (19.8%)
Anaesthetic complication	360 (16.9%)
ASA score	2.1 (SD: 0.9)
RCI	318 (SD: 193)
Staffing level	3 (SD: 1)
Total duration (mins)	235 (SD: 137)
Preparation	15 (SD: 4)
Installation	24 (SD: 11)
Anaesthesia	27 (SD: 18)
Intervention	123 (SD: 111)
Dressing	8 (SD: 4)
Cleaning	20 (SD: 11)
Top-down cost (Euros)	2 331 (SD: 1 720)
Bottom-up cost (Euros)	2 186 (SD: 1 391)

Data shown are mean (SD) or N (%); costs are in Euros.

**Table 2:** Activity duration, cost drivers, value of unit costs and costs (staff costs only).

Activity	Duration	Cost driver	Unit cost	Total cost
Individual setup	36 715	minute	4.87	178 649
Patient’s positioning	62 815	minute	3.82	239 881
Anaesthesia induction	55 360	minute	5.92	327 751
Intervention	283 636	minute	7.17	2 033 494
Intervention (surgeon)	923 673	minute*nsurgeons	0.30	273 422
Wound dressing	19 794	minute	2.47	48 796
Cleaning	46 476	minute	4.87	226 220
Recovery	NA	volume	89.55	263 557
Daily setup	NA	volume	74.06	192 264
Planning	NA	volume	8.79	22 810
Surgical management	NA	volume	76.51	198 609
Anaesthetic management	NA	volume	39.58	116 476
Total	NA	NA	NA	4 121 928

All durations are in minutes; costs are in Euros.

**Figure 1:**
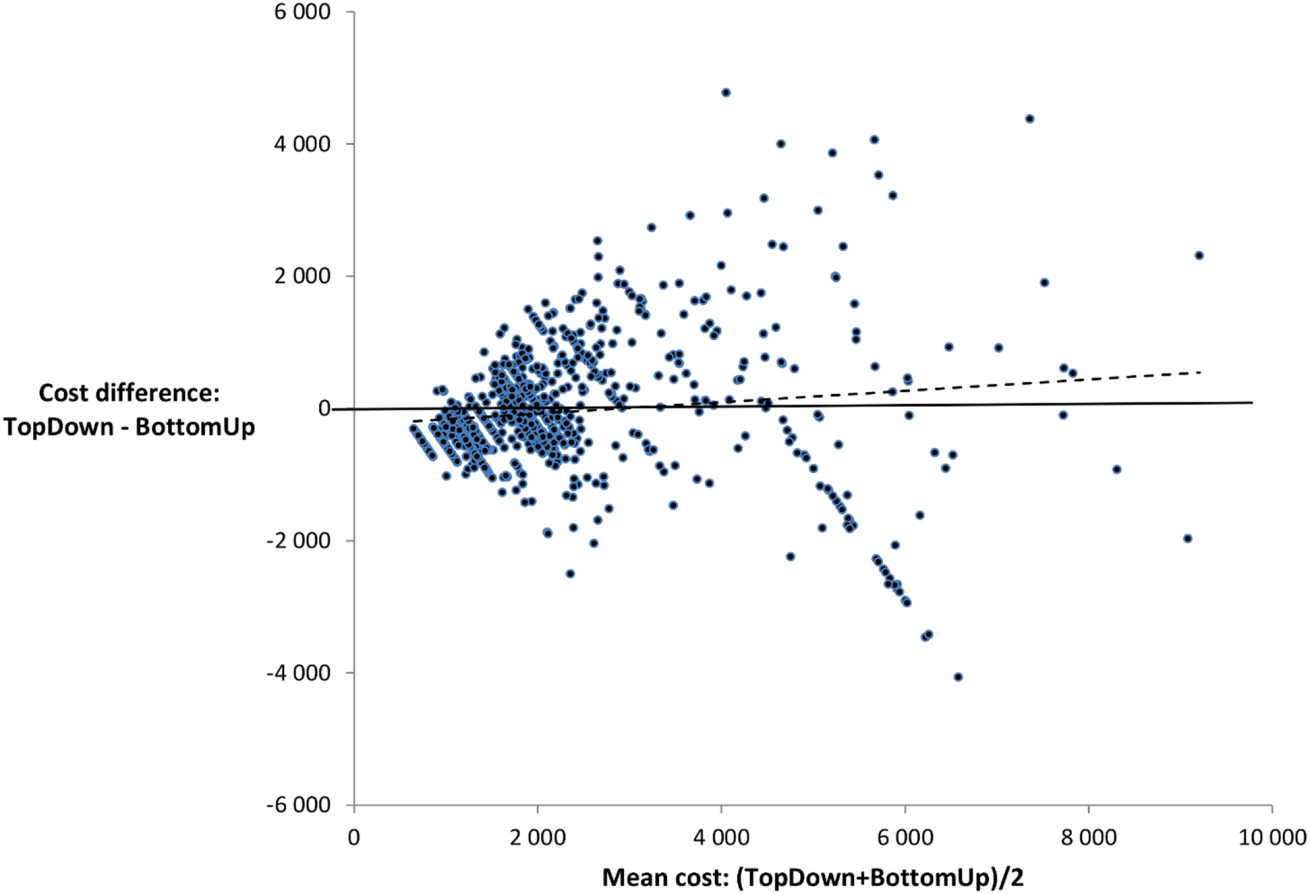
Bland and Altman plot of the difference between the costing methods against their mean. Each dot represents an individual procedure; the dotted line shows the ordinary least squares regression.

The Spearman non-parametric coefficient of correlation amounted to 0.73 (95%CI: 0.72; 0.76), which is adequate for a group comparison but not for an individual assessment. The mean difference between bottom-up and top-down costs was 144 Euros (SD: 1 168). The Bland and Altman graph shows an overall poor agreement between the two costing methods, as the vast majority of points lie above or below the central line. The lower and upper agreement limits are equal to −2 146 and 2 434 Euros, respectively (Figure 1). There is no obvious fixed bias but rather a clear proportionate one: the size of the difference between the methods increases with the mean cost.

The bivariate analysis suggested that the cost difference was positively associated with age, female sex, ASA score, RCI and staffing level and was negatively associated with junior surgeon and intervention duration (Table 3). Thus, variables included in the multivariate model were age, sex, junior surgeon, ASA score, RCI, staffing level and intervention duration. In the multivariate analysis, the cost difference was independently associated with age (Beta = −2.4; p = 0.02), ASA score (Beta = 76.3; p<0.001), RCI (Beta = 5.5; p<0.001), staffing level (Beta = 437.0; p<0.001) and intervention duration (Beta = −10.5; p<0.001). Sex and surgeon status were not significantly associated with cost difference. As the cost difference was calculated as the top-down cost minus the bottom-up cost, the former cost was significantly higher than the latter when patients were female and had a higher anaesthetic risk, when the RCI of the procedure was higher and when the number of surgeons (staffing level) was higher. By contrast, the top-down cost was significantly lower when the patient was older and when the intervention phase was longer. On average, a one year increase in age was associated with a 2.4 Euros decrease in cost difference; a one point increase in the ASA score was associated with a 76.3 Euros increase in the difference; a one point increase in the procedure RCI was associated with a 5.5 Euros increase; and a one minute increase in the duration of the intervention phase was associated with a 10.5 Euros decrease. The model R-square was equal to 0.63.

**Table 3:** Variables associated with the cost difference in bivariate and multivariate analysis (n = 2130).

	Bivariate analysis			Multivariate analysis		
Variable	Beta	SE	p-value	Beta	SE	p-value
Age (years)	4.9	1.4	<0.001	−2.4	1.0	0.02
Female patient	208.2	50.6	<0.001	21.7	32.2	0.50
On call period	−112.6	80.3	0.16	--		
Emergency procedure	−27.0	63.5	0.67	--		
Anaesthetic complication	24.7	67.6	0.7	--		
Junior surgeon	−414.0	157.9	0.01	−65.6	103.9	0.53
ASA score	85.8	27.7	0.002	76.3	20.0	<0.001
RCI	2.1	0.1	<0.001	5.5	0.1	<0.001
Staffing level	434.2	18.4	<0.001	437.0	14.8	<0.001
Intervention duration	−1.2	0.2	<0.001	−10.5	0.2	<0.001

Beta: regression coefficient. SE: Standard Error. Variables included in the model were age, sex, junior surgeon, ASA score, RCI, staffing level and intervention duration.

## Discussion

This study aimed to investigate the sensitivity of the patient-level cost of surgical services to the costing method. On a consecutive sample of 2 130 surgical procedures performed in a French tertiary centre, the overall agreement between top-down and bottom-up microcosting appears to be poor. Indeed, the correlation coefficient amounted to 0.73 (95%CI: 0.72; 0.76), which might be sufficient for group comparisons (value>0.50) as part of a cost-effectiveness analysis but not for patient-level analysis (value<0.90). According to the Bland and Altman method, the upper and lower limits of agreement both exceed 2 000 Euros in absolute value, which means that 95% of the true differences between the microcosting methods would be lower than that amount. Given the average unit cost at Montpellier University Hospital in 2010 (2 052 Euros), this amount is obviously substantial. These findings corroborate previous work highlighting the sensitivity of inpatient cost estimates to the costing method [9], [11], [13], [14]. We focused on surgery services, while most published studies do not distinguish between surgery and non-surgery services. Nonetheless, our results strongly support the fact that bottom-up microcosting significantly departs from top-down methods in labour-intensive services [10].

In the multivariate analysis, the cost difference between methods was independently associated with age, ASA score, procedure RCI, staffing level and intervention duration. The magnitude is particularly high regarding procedure RCI (Beta = 5.5) and intervention duration (Beta = −10.5). Indeed, a one-standard deviation increase in the RCI or in the intervention duration would translate to a 1 061 Euros increase or a 1 165 decrease, respectively, in the cost difference. Staffing level and intervention duration were used in the bottom-up costing method, while the procedure RCI was the main cost driver in the top-down method. All three drivers are somehow related to the complexity of surgical procedures. However, RCIs are cost weights defined a priori to reflect average resource consumption levels [30]. As RCIs are constant for a given surgical procedure, they do not account for intra-procedure variations due to patient-level factors. In this study, the duration might reflect surgery complexity as it excludes in-operating room waiting times, but the relationship is not straightforward. Indeed, the intervention duration is strongly linked with surgery complexity, and duration and complexity are sometimes considered to be the same concept [33]. The staffing level can be an indirect marker of surgery complexity and a marker of organisational performance [34]. Age and ASA score reflect surgery complexity related to underlying patient-level factors. Patient sex, surgeon status, on-call period, emergency context and anaesthetic complications were not significantly associated with the cost difference. This outcome might be due to collinearity and to a lack of power. For instance, the ASA score is strongly associated with the risk of anaesthetic complication, and there were only 56 (2.6%) procedures performed by junior surgeons.

The methods compared in this work are both microcosting approaches, but the bottom-up approach is more accurate at the patient level [5], [7], [10]. Indeed, using patient-level factors such as actual duration and staffing level allows the cost to reflect individual resource consumption variations [21]. Consequently, the ABC bottom-up cost can be taken for a proxy of the true opportunity cost, and any deviation from this cost might be interpreted as a cross subsidisation. The current top-down microcosting method tends to overestimate the cost of procedures performed on patients with a high ASA score, involving more staff and having a high RCI. Nevertheless, this method underestimates the cost of longer procedures performed on older patients. These potentially conflicting findings might be partially explained by the fact that staffing levels and RCI are poorly associated with surgical complexity [30], [34]. However, these assumptions are not easily testable and must therefore be considered cautiously.

These results might be analysed considering that the top-down method allocates indirect charges only based on the RCI. Hence, this method does not have the same ability to reflect patient-level factors. This situation reinforces the hypothesis that the top-down method imperfectly captures the complexity of procedures [20].

Altogether, the agreement between bottom-up and top-down microcosting is poor, bottom-up costs reveal cross-subsidisation, and the difference between both methods is explained by patient-level factors.

These findings have potential implications for hospital transparency and efficiency.

In most high and middle income countries, hospitals are increasingly paid through prospectively defined DRGs tariffs, which are usually based on national average costs calculated on a sample of volunteer hospitals [1]. One of the underlying assumptions is that cost variation between hospitals reflects heterogeneity in endogenous factors over which hospitals have control. If not, some DRGs may be artificially more profitable than others, and the allocative efficiency of the system will be threatened [35]. Our results suggest that some patient-level cost factors are heterogeneous, namely the individual anaesthetic risk level (ASA score) and age. These factors are not reflected by the top-down service costs. If the distribution of these non-controllable factors is not equal across hospitals, then the fairness and efficiency of the financing system are questionable.

This study suggests that top-down costing methods do not reflect some important potentially controllable factors such as medical and nursing staffing levels and, to some extent, intervention duration. Consequently, hospitals might fail to identify and target inefficient processes.

An improvement could be to shift from the current top-down costing method towards a bottom-up method in France. As activity-based costing in the hospital setting is considerably resource-consuming [14], implementing a stepwise strategy towards a long-term objective of time-driven activity-based costing is a more realistic option [5]. Regarding surgery services, one crucial change is to allocate operating-room charges using surgery and anaesthesia durations instead of pre-defined weights such as RCI. In doing so, the French method would partly converge towards other European hospital costing systems such as the English and the German systems [5].

This study suffers several limitations. A large and exhaustive sample of operations was included but within a single surgery department in a single hospital. This approach could hinder the generalisability of our findings. Nevertheless, it is plausible that allocating staff costs to individual medical procedures entails the same steps and the same issues no matter the setting. Consequently, the poor agreement between top-down and bottom-up costing methods might be generalisable to a certain extent. As described in previous papers reporting the implementation of ABC models, the cost of data capture and analysis is very high [27]. However, our method relates to time-driven activity-based costing (TD-ABC) in that most of the activity and resource drivers are durations [36]. TD-ABC has been shown to be less resource-intensive than ABC [37], and our work might therefore be more easily generalisable to other settings. In the activity-based costing, non-staff costs have been allocated proportionally to staff costs, which might not necessarily reflect the actual resource consumption. Nevertheless, we have done so in order to be consistent with the top-down costing method, and our approach tends to reduce any difference between the two methods.

## Conclusion

Accurate patient-level costing is critical to improve efficiency and transparency in the hospital setting. Based on a large sample of consecutive surgical procedures in a French tertiary centre, this study confirms the overall poor agreement between top-down and bottom-up methods. The current top-down method fails to reveal patient-level resource-use variations and leads to considerable cross-subsidisations. Hence, the ability of the current method to provide relevant information to managers, clinicians and payers is questionable. As in other European countries, a shift towards time-driven activity-based costing should be advocated.
